# Telerehab III: a multi-center randomized, controlled trial investigating the long-term effectiveness of a comprehensive cardiac telerehabilitation program - Rationale and study design

**DOI:** 10.1186/s12872-015-0021-5

**Published:** 2015-05-07

**Authors:** Ines Frederix, Dominique Hansen, Karin Coninx, Pieter Vandervoort, Emeline M. Van Craenenbroeck, Christiaan Vrints, Paul Dendale

**Affiliations:** Department of Cardiology, Jessa Hospital, Stadsomvaart 11, 3500 Hasselt, Belgium; Faculty of Medicine & Life Sciences, Hasselt University, Agoralaan gebouw D, 3590 Diepenbeek, Belgium; Faculty of Sciences, Expertise Center for Digital Media, Hasselt University, Wetenschapspark 2, 3590 Diepenbeek, Belgium; Department of Cardiology, Hospital East-Limburg, Schiepse Bos 6, 3600 Genk, Belgium; Department of Cardiology, Antwerp University Hospital, Wilrijkstraat 10, 2650 Edegem, Belgium

**Keywords:** Cardiac telerehabilitation, Cardiovascular disease, Intervention trial, Telemonitoring and telecoaching

## Abstract

**Background:**

Telerehabilitation has been proposed as an adjunct/alternative to standard center-based cardiac rehabilitation. Two recent systematic reviews showed non-inferiority and/or superiority of this remote approach for cardiac rehabilitation. However, these trials focused only on one core component of cardiac rehabilitation and telemonitoring, rather than implementing a more comprehensive approach. The aim of Telerehab III is to investigate the long-term effectiveness of the addition of a patient-tailored, internet-based telerehabilitation program implementing multiple cardiac rehabilitation core components and using both telemonitoring and telecoaching strategies to standard cardiac rehabilitation.

**Methods/Design:**

In this prospective, multi-center randomized, controlled trial 140 patients with coronary artery disease and/or chronic heart failure patients will be recruited between February 2013 and February 2015. Patients will be randomized 1:1 to an intervention group (receiving an internet-based telerehabilitation program in addition to standard cardiac rehabilitation) or to standard cardiac rehabilitation alone. The mean follow-up is at least 6 months. The primary endpoint is peak oxygen consumption (VO2 peak). Secondary endpoints include measured and self-reported daily physical activity, cardiovascular risk factor control, health-related quality of life, days lost due to (non)cardiovascular rehospitalizations and time to first (non)cardiovascular rehospitalization. A clinical event committee blinded to treatment allocation assesses causes of rehospitalizations.

**Discussion:**

Telerehab III will be one of the first studies to examine the added value of a more comprehensive cardiac telerehabilitation program, focusing on multiple cardiac rehabilitation core components. It has the potential to augment current standard center-based cardiac rehabilitation practices and to be used as a model for other disease prevention programs.

**Trial registration:**

Current controlled trials ISRCTN29243064. Registration date 21 January 2015.

## Background

Secondary prevention of cardiovascular disease (CVD) by means of cardiac rehabilitation (CR) is a Class IB recommendation by the European Society of Cardiology (ESC), the American Heart Association (AHA) and the American College of Cardiology [1]. It is comprised of different core components such as (increase in) physical activity, behavioural change, risk factor modification, nutritional counselling and stimulation of psychosocial wellbeing [2, 3]. However, despite the proven clinical effectiveness of conventional supervised center-based CR (improvements in CVD risk factors, physical activity and physical fitness) [4], long-term benefits are often disappointing due to lack of adherence and persistent change in lifestyle [5, 6]. In addition, patients regularly choose not to attend in-hospital rehabilitation sessions due to a lack of access to transport, ill-health, time and scheduling commitments associated with returning to work or reimbursement issues. It thus follows that suboptimal secondary CVD prevention emerges. Therefore, new strategies or innovations in adjunct/addition to CR, that would lead to increased participation rates and long-term therapy adherence, are urgently warranted.

Recent technological innovations in ICT can be a potential adjunct and/or alternative to enhance rehabilitation adherence and hence long-term effectiveness of CR programs. In telerehabilitation and -prevention, the patient is not restricted to the hospital or rehabilitation center environment for CR, but rehabilitates remotely by using one or several devices monitoring and communicating patient-specific information to the caregivers.

Two recent systematic reviews regarding cardiac telerehabilitation programs showed non-inferiority and/or superiority of this approach, when compared to conventional center-based supervised CR [7, 8]. However, in most studies only one or two of the CR core components (physical activity, behavioural change, risk factor modification, nutritional counselling and psychosocial wellbeing) were integrated in the studied intervention. Telemonitoring and telecoaching were the two focus areas mostly applied, the use of combined approaches and tailoring to the patients’ needs was limited. This demonstrates the need for the implementation of a more comprehensive telerehabilitation program.

The Telerehab III trial is a randomised, controlled, multi-center study designed to investigate the long-term effectiveness of the addition of a patient-tailored, internet-based telerehabilitation program implementing multiple CR core components and using both telemonitoring and telecoaching strategies to conventional CR; when compared to conventional center-based CR alone. The primary hypothesis is that the addition of telerehabilitation increases patient’s physical fitness and activity level with greater magnitude, when compared to usual care. Secondary endpoints include cardiovascular risk factor control, quality of life and (non-) cardiovascular rehospitalizations.

## Methods/Design

### Study design

**Fig. 1:**
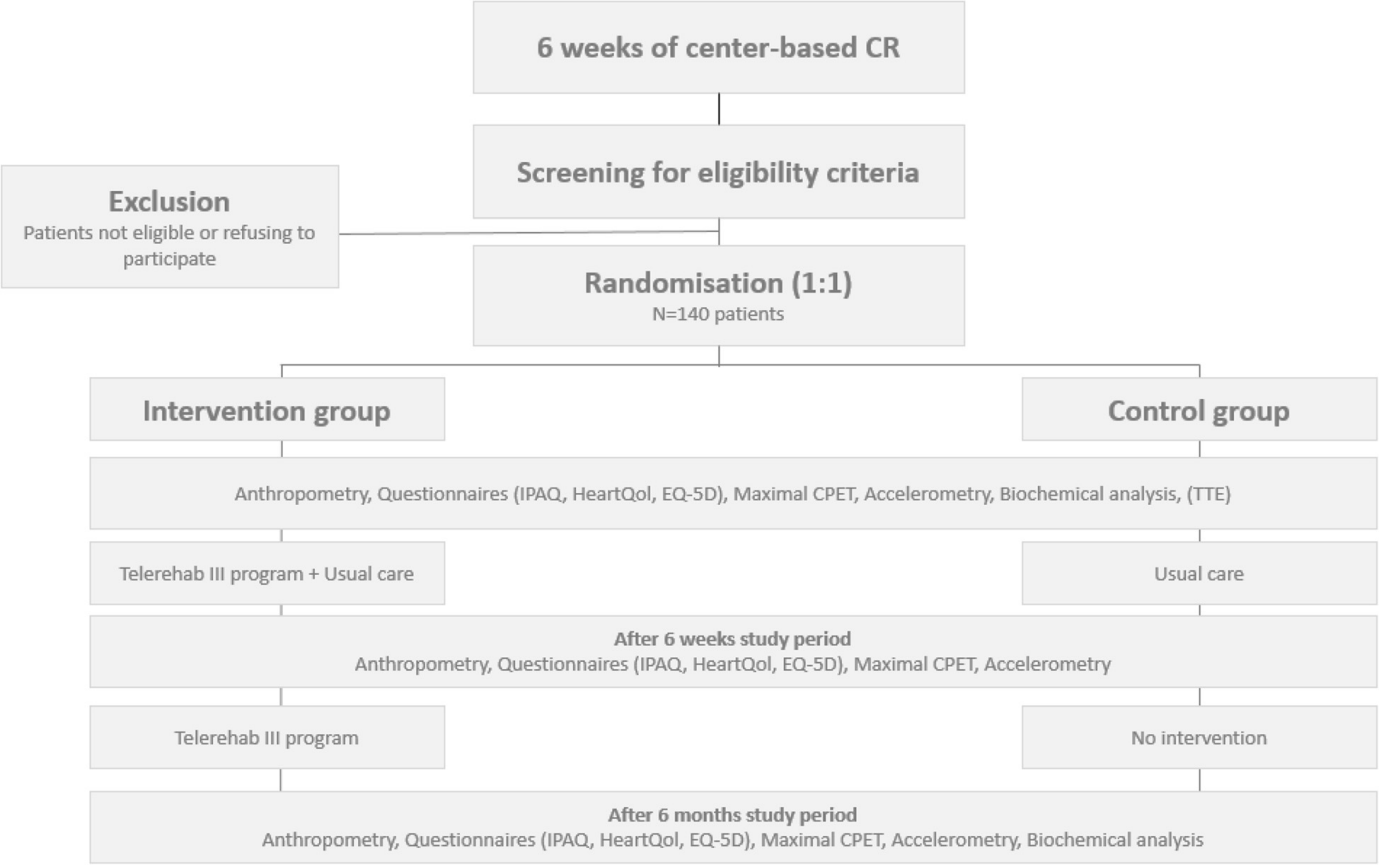
Flow chart for patient inclusion and follow-up. Screening, inclusion and exclusion, randomization and tests at baseline and follow-up. CR: Cardiac Rehabilitation; IPAQ: International Physical Activity Questionnaire; HeartQol: quality of life questionnaire; EQ-5D: EuroQol questionnaire; CPET: CardioPulmonary Exercise Testing; TTE: TransThoracic Echocardiography

Telerehab III is a multi-center, prospective, randomized, controlled clinical trial recruiting one hundred and forty stable coronary artery disease (CAD) and/or chronic heart failure (CHF) patients (Fig. 1). Patients are randomized in equal proportions to one of two groups: internet-based telerehabilitation in addition to center-based rehabilitation (intervention group) or center-based rehabilitation alone (control group). Patients are enrolled after completion of the first six weeks of their phase II center-based rehabilitation program. They are followed for a minimum of 6 months and a maximum of 25 months.

Patients are recruited from the Cardiology Departments of three Belgian hospitals i.e. Jessa Hospital (Hasselt), Ziekenhuis Oost-Limburg (Genk) and St. Franciscus Hospital (Heusden-Zolder). The Jessa Hospital acts as the coordinating center for the trial. Each participating site is responsible for the recruitment and scheduled follow-up visits of patients; but also for contingency patient management. Each site recruits at least ten patients.

The study is conducted in accordance with the principles stated in the Declaration of Helsinki (reviewed version of 2008), local and national regulations. Written informed consent is obtained from all patients prior to study enrollment. The study is approved by the appropriate ethics committee prior to study enrollment, (Jessa Ethics Committee; reference number: B243201216043).

The study adheres to the relevant standards of reporting. A completed CONSORT checklist can be found in Table 1. There were no important changes to the methods after trial commencement.

**Table 1:** Completed CONSORT checklist for Telerehab III

Section/Topic	Item No	Reported in section
Title and abstract		
	1a	
	1b	See abstract. No results and conclusions yet since study design paper
Introduction		
Background and objectives	2a	See Background
	2b	See Background
Methods		
Trial design	3a	See Methods (Study design)
	3b	See Methods (Study design)
Participants	4a	See Methods (Study population)
	4b	See Methods (Study design)
Interventions	5	See Methods (Study intervention)
Outcomes	6a	See Methods (Study endpoints)
	6b	See Methods (Study endpoints)
Sample size	7a	See Methods (Sample size)
	7b	See Methods (sample size)
Randomization:		
Sequence generation	8a	See Methods (Randomization)
	8b	See Methods (Randomization)
Allocation concealment mechanism	9	See Methods (Randomization)
Implementation	10	See Methods (Randomization)
Blinding	11a	See Methods (Study endpoints)
	11b	Not applicable
Statistical methods	12a	See Methods (Statistical analysis)
	12b	See Methods (Statistical analysis)
Results		
Participant flow	13a	Not yet available (since study design paper)
	13b	Not yet available (since study design paper)
Recruitment	14a	See abstract
	14b	Not applicable
Baseline data	15	Not yet available (since study design paper)
Numbers analysed	16	Not yet available (since study design paper)
Outcomes and estimation	17a	Not yet available (since study design paper)
	17b	Not yet available (since study design paper)
Ancillary analyses	18	Not yet available (since study design paper)
Harms	19	Not yet available (since study design paper)
Discussion		
Limitations	20	Not yet available (since study design paper)
Generalisability	21	Not yet available (since study design paper)
Interpretation	22	Not yet available (since study design paper)
Other information		
Registration	23	See Trial registration
Protocol	24	See Main manuscript
Funding	25	See Acknowledgements

### Study population

Patients belonging to one of the two cardiac disease categories (ia CAD for which patients were treated conservatively, with a percutaneous coronary intervention or with coronary artery bypass grafting OR ib CHF with reduced EF (NYHA I, II and III) OR ic CHF with preserved EF (NYHA I, II and III)) and who had signed the informed consent form; are eligible to participate. All patients were on optimal medical treatment and stable for >4 weeks. In addition, eligible patients are at least 18 years and maximally 80 years old. They must have a personal computer with internet connection.

The main exclusion criteria are: the presence of a pre-existing non-cardiovascular condition (orthopedic and/or neurological), limiting the patient’s ability to actively engage in exercise training; the presence of terminal disease, dementia and cognitive impairment that unables the patient to use the telerehabilitation equipment or appear to follow-up visits. The complete list of inclusion and exclusion criteria is provided in Table 2.

**Table 2:** Summary of key inclusion and exclusion criteria for Telerehab III

Inclusion criteria
ia. CAD patients treated conservatively, with PCI or CABG.
ib. CHF patients with reduced EF
ic. CHF patients with preserved EF
ii. Current active rehabilitation on one of the recruiting centres
iii. Possesion of personal computer with internet connection
iv. Age >18 and <80 years
v. Familial with Dutch language
vi. Informed consent
Exclusion criteria
i. Orthopaedic and/or neurological condition, limiting the patient’s ability to actively engage in exercise training sessions
ii. Impairment to use the telerehabilitation equipment or appear at follow-up visits (terminal disease, dementia and cognitive impairment)
iii. Simultaneous participation in anoter clinical trial
iv. Patients with CHF NYHA IV
v. Patients with a history of VF. exertional sustained VT/supraventricular tachycardia within the previous 6 months

*CAD* coronary artery disease, *CHF* chronic heart failure, *PCI* percutaneous coronary intervention, *CABG* coronary artery bypass grafting, *EF* ejection fraction, *NYHA* New York Heart Association, *VF* ventricular fibrillation, *VT* ventricular tachycardia

### Sample size

The sample size calculation is based on a 20 % effect size of VO2 peak , which is comparable to the effect reported by Frederix et al. in their Telereab II study [9]. In order to attain a minimal power of 95 %, at an alpha error probability < 0,05; 140 patients should be recruited when taking into account a dropout rate of 30 % during follow-up.

### Randomization

Patients are assigned to one of two treatment arms by a central computerized randomization system. Block randomization is used to ascertain equal distribution of patients in the intervention and control group in the different recruiting hospitals. In order to achieve a balance of relevant patient characteristics in both study arms, it will be assessed whether patients are matched for age, gender, cardiac disease category and cardiovascular risk factor profile. When intervention and control patients are not matched for these characteristics, a second randomization process will be done to ascertain matching. Investigators are unaware of the randomization sequence.

### Outcome assessments

At entry of study a complete medical history, clinical assessment (including measurement of body mass index, waist circumference, blood pressure and heart rate) and treatment scheme are collected. A transthoracic echocardiography (TTE) evaluating LV systolic function (using Simpson’s method and/or eye balling), LV diastolic function (E/A ratio, DT, IVRT and pulmonary venous flow curves) and presence/absence of increased left atrial filling pressures (average E/E’) is planned, when no recent TTE (performed < 6 months earlier) is available. Every patient performs a maximal cardiopulmonary exercise test (CPET) with breath-by-breath gas exchange analysis. The criteria for defining maximal CPET are an achieved heart rate > 85 % of the maximal predicted heart rate, an achieved Respiratory Exchange Ratio (RER) > 1,1, and/or achieving a plateau on the VO2 curve. During CPET, we will record RER, resting and maximal heart rate, resting and maximal systolic and diastolic blood pressure, resting and maximal rate pressure product, VO2 peak, maximal work load, ventilatory anaerobic threshold, respiratory compensation point and the oxygen uptake efficiency slope. We will use the recommendations provided by Wasserman et al. to predict VO2 peak and to calculate % of predicted VO2 peak [10]. Fasting blood samples are taken, to determine blood glucose, HbA1c, CRP level and lipid profile. Patients are asked to complete a quality of life questionnaire (HeartQol), a questionnaire to assess self-reported physical activities (IPAQ) and the EQ-5D questionnaire. After randomization, study visits were scheduled after 6 weeks as well as at the end of study period. During these visits, clinical assessment, treatment scheme collection and maximal CPET are performed. Patients are again asked to complete the HeartQol, IPAQ and EQ-5D questionnaires. Blood sampling is only repeated after study completion (Fig. 1).

### Study intervention

Patients in the intervention group are provided with an internet-based telerehabilitation program in addition to conventional center-based CR. The telerehabilitation program is composed of physical activity telemonitoring (Yorbody accelerometer) in addition to dietary, smoking cessation and activity telecoaching.

#### The telerehabilitation service

Study nurse led training is given to patients within 7 days after randomization. They are provided with some background and general information regarding the study design, content and main hypotheses of Telerehab III. Patients are taught about how to install the Telerehabilitation program on their computer and how to use the accelerometer. This presentation is followed by a one hour lasting practical training session in which patients are familiarized with the accelerometer and associated webpage. They are instructed to install the program at home; however in case of questions or technical difficulties, they are provided with technical support.

#### The exercise training protocol

**Fig. 2:**
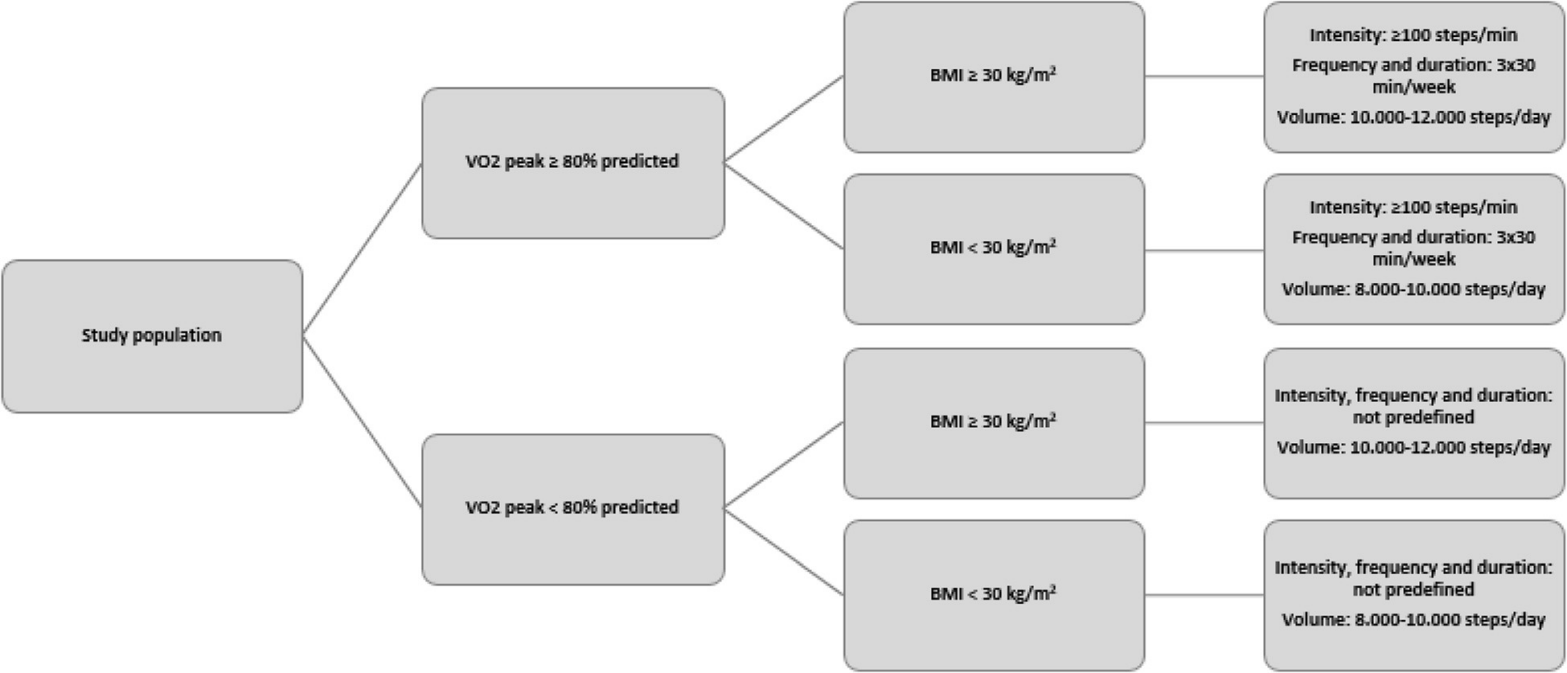
Algorithm defining intensity, frequency, duration and volume characteristics of exercise training protocols used in the telerehabilitation program

Intervention patients are prescribed with literature based [11–13], patient-specific exercise training protocols. These exercise protocols are based on maximal CPET and calculated body mass index (BMI) at the start of study period. Patients with a high aerobic capacity (defined as VO2 peak ≥ 80 % predicted) are stimulated to exercise at an intensity of ≥ 100 steps/min, for at least 3 times/week and minimal session duration of 30 min; in order to increase the likelihood for significant changes in exercise tolerance [14]. Patients with a low aerobic capacity (defined as VO2 peak < 80 % predicted) were allowed to choose themselves the intensity of exercise sessions. In addition, volume of exercise depended on body mass index: The instructed volume of steps was set at 10.000-12.000 steps/day for patients with a BMI > 30 kg/m2 and 8.000-10.000 steps/day for patients with a BMI < 30 kg/m2 (Fig. 2).

Patients are instructed to wear the accelerometer during entire study period, except while bathing and/or sleeping. Due to the accelerometer’s storage capacity of 14 days, patients upload data at least every 2 weeks to a secure webpage with an USB-connection. The webpage displays uploaded physical activities thereby enabling patients to self-monitor these data. Based on the uploaded data a semi-automatic telecoaching system, provides patients with weekly feedback via e-mail and/or SMS. This feedback is intended to encourage patients to achieve the goals as predefined in the patient-specific exercise training protocols.

Uploaded accelerometry data are automatically transmitted in an encrypted manner to the telerehabilitation’s local server. All procedures are in compliance with confidentiality standards for medical data (De Wet op het Privéleven met Betrekking tot de Behandeling van Persoonlijke gegevens [W 1998-12-11/54]). Authorized medical staff treating the patient is granted unconstrained access to the patients’ data, whereas restricted access to anonymized data is granted to other local staff and researchers.

#### The dietary and smoking cessation telecoaching program

Intervention patients receive weekly dietary and smoking cessation advice, sent by email and/or SMS according to the patients’ preferences. The dietary telecoaching program includes a module for diabetes mellitus, for arterial hypertension, for obesity and a healthy module. Cardiovascular risk factor profiling at entry of study determines which module(s) are prescribed for each patient. Key to the diabetes mellitus module is the restriction of fast carbohydrates (monosaccharides), energy rich nutrition and ethanol containing beverages. The arterial hypertension and obesity modules focus on salt restriction (<5 g/day) and energy intake reduction respectively. Healthy diet includes recommendations based on the food triangle [15]. The smoking cessation telecoaching program includes information on major risks associated with smoking, the health benefits of smoking cessation and nicotine replacement therapy. It provides smokers with encouraging messages towards smoking cessation.

### Conventional cardiac rehabilitation care

Patients in the control group follow the conventional center-based CR program; including 45 pluridisciplinary rehabilitation sessions focusing on healthy diet, psychosocial wellbeing, behavioural change, risk factor modification and exercise training. The exercise training sessions are scheduled 2 to 3 times/week; with an average session duration of 45 min. Training intensity is progressively increased from the ventilatory anaerobic threshold to the respiratory compensation point. Main exercise modalities include walking/running and cycling.

These patients are instructed to wear the accelerometer three times; at study start, after 6 weeks and at the end of study period for observation purposes only. The sensors are taped at the hospital using black tape, making it impossible for the patient to see the registered physical activity data. Data uploading is performed by study nurses of the respective hospitals, patients cannot consult the webpage with uploaded data. They do not receive physical activity, smoking cessation or dietary telecoaching by email and/or SMS.

### Study endpoints

The primary endpoint is peak oxygen consumption (VO2 peak). The first secondary endpoint is daily physical activity, defined as the total number of daily low intensity and high intensity steps.

Other secondary endpoints include:

- Cardiovascular risk factor control (body weight, blood pressure, blood lipid profile, blood glucose level and HbA1c) at 6 weeks and 6 months
- HeartQol quality of life score at 6 weeks and 6 months, adjusted for baseline
- IPAQ physical activity score at 6 weeks and 6 months, adjusted for baseline
- EQ-5D score at 6 weeks and 6 months, adjusted for baseline
- Days lost due to cardiovascular rehospitalization
- Days lost due to hospitalization for any reason
- Time to first cardiovascular rehospitalization
- Time to first hospitalization for any reason.

Outcome assessors were blinded to treatment allocation.

### Clinical Endpoint Committee

A Clinical Endpoint Committee (CEC), composed of three specialists in internal medicine (with at least two of them cardiologists), will classify all rehospitalizations. This classification will be based on the completion of templates defining the criteria and definitions for cardiovascular and/or non-cardiovascular rehospitalizations.

### Statistical analysis

The statistical analysis will be performed according to the Intention-to-treat principle. For continuous data, the Shapiro-Wilk test will investigate whether normal distribution is present. When data are not normally distributed, the Mann–Whitney U-test will be used for the comparison between groups; and the Wilcoxon Signed Rank test will be used for the comparison within groups. When data are normally distributed the One Way ANOVA repeated measures test will be used for both between and within group analysis. Kaplan-Meier plots and log-rank tests will be used to compare censored time-to-event data; for the time to first cardiovascular rehospitalization and time to first rehospitalization for any reason. The statistical software package SPSS will be used for analysis. P-values <0,05 will be considered statistically significant.

## Discussion

The Telerehab III trial investigates the long-term effectiveness of the addition of a patient-tailored, internet-based telerehabilitation program implementing multiple CR core components and using both telemonitoring and telecoaching strategies to conventional CR.

Several clinical trials on cardiac telecare have recently been published, including the TIM-HF trial [16], the Telerehab II trial [9], the CHOICE trial [17–19], the COACH trial [20, 21], the eOCR trial [22, 23], and the TeleInterMed trial [24]. Hence two systematic reviews have recently been published regarding the feasibility, efficacy, safety and cost-effectiveness of cardiac telecare [7, 8].

The first review reported on cardiac telerehabilitation in CAD and CHF patients with a total of 13.248 patients enrolled in 37 studies; with a mean follow-up period of 9 months [7]. The review showed that telerehabilitation was associated with significantly favouring results for the tele-intervention regarding adherence to physical activity guidelines (Odds Ratio (OR) = 0,56, 95 % CI: 0,45-0,69); and the combined endpoint of adverse events and cardiovascular rehospitalizations (OR = 1,30, 95 % CI: 1,13-1,50). In the systematic review of Huang *et al.* nine trials of patients with CAD were reviewed. No statistically significant difference was found between telehealth interventions and center-based supervised CR in exercise capacity (standardized mean difference (SMD) = −0,01; 95 % CI: −0,12-0,10), body weight (SMD = −0,13; 95 % CI: −0,30-0,05), systolic and diastolic blood pressure (mean difference (MD) = −1,27; 95 % CI: −3,67-1,13 and MD = 1,00; 95 % CI: −0,42-2,43, respectively), blood lipid profile, smoking behaviour (risk ratio (RR) = 1,03; 95 % CI: 0,78-1,38), mortality (RR = 1,15; 95 % CI: 0,61-2,19), quality of life and psychosocial state.

Both reviews provide strong arguments for the non-inferiority and/or superiority of investigated telerehabilitation programs; when compared to standard center-based supervised CR. However, contrary to clinical trials included in these reviews, Telerehab III will be one of the first studies to prospectively test the long-term effectiveness of a patient-specific, multi core component cardiac telerehabilitation intervention; focusing both on telemonitoring and telecoaching. This comprehensive approach will have the ability to further improve effectiveness of tele-intervention based programs.

## Trial status

The study protocol and amendments were approved by the hospitals’ ethics committee prior to study start (B243201216043). The study is currently ongoing.

## Abbreviations

BMI: Body mass index
CABG: Coronary artery bypass grafting
CAD: Coronary artery disease
CEC: Clinical endpoint committee
CHF: Chronic heart failure
CPET: Cardiopulmonary exercise testing
CR: Cardiac rehabilitation
CVD: Cardiovascular disease
EF: Ejection Fraction
IPAQ: International physical activity questionnaire
NYHA: New York Heart Association
PCI: Percutaneous coronary intervention
RER: Respiratory exchange ratio
TTE: Transthoracic echocardiography
VF: Ventricular fibrillation
VT: Ventricular tachycardia
